# A Rodent Model of Human-Dose-Equivalent 5-Fluorouracil: Toxicity in the Liver, Kidneys, and Lungs

**DOI:** 10.3390/antiox12051005

**Published:** 2023-04-26

**Authors:** Mariana Conceição da Silva, Lilian Catarim Fabiano, Karile Cristina da Costa Salomão, Pedro Luiz Zonta de Freitas, Camila Quaglio Neves, Stephanie Carvalho Borges, Maria das Graças de Souza Carvalho, Ana Cristina Breithaupt-Faloppa, André Alexandre de Thomaz, Aline Mara dos Santos, Nilza Cristina Buttow

**Affiliations:** 1 Biological Physics and Cell Signaling Laboratory, Institute of Biology, Department of Structural and Functional Biology, State University of Campinas, Campinas 13083-970, SP, Brazilm219793@dac.unicamp.br (M.d.G.d.S.C.); 2 Department of Morphological Science, State University of Maringá, Maringá 87020-900, PR, Brazil; 3 Departament of Biological Science, State University of Santa Cruz, Ilhéus 45662-900, BA, Brazil; 4 Laboratório de Cirurgia Cardiovascular e Fisiopatologia da Circulação (LIM-11), Instituto do Coração (InCor), Faculdade de Medicina da Universidade de São Paulo, São Paulo 01246-904, SP, Brasil; 5 Quantum Electronic Department, Institute of Physics Gleb Wataghin, State University of Campinas, Campinas 13083-872, SP, Brazil

**Keywords:** cytokines, histology, chemotherapy, adverse effects, oxidative stress

## Abstract

5-Fluorouracil (5-FU) is a chemotherapy drug widely used to treat a range of cancer types, despite the recurrence of adverse reactions. Therefore, information on its side effects when administered at a clinically recommended dose is relevant. On this basis, we examined the effects of the 5-FU clinical treatment on the integrity of the liver, kidneys, and lungs of rats. For this purpose, 14 male Wistar rats were divided into treated and control groups and 5-FU was administered at 15 mg/kg (4 consecutive days), 6 mg/kg (4 alternate days), and 15 mg/kg on the 14th day. On the 15th day, blood, liver, kidney, and lung samples were collected for histological, oxidative stress, and inflammatory evaluations. We observed a reduction in the antioxidant markers and an increase in lipid hydroperoxides (LOOH) in the liver of treated animals. We also detected elevated levels of inflammatory markers, histological lesions, apoptotic cells, and aspartate aminotransferase. Clinical treatment with 5-FU did not promote inflammatory or oxidative alterations in the kidney samples; however, histological and biochemical changes were observed, including increased serum urea and uric acid. 5-FU reduces endogenous antioxidant defenses and increases LOOH levels in the lungs, suggesting oxidative stress. Inflammation and histopathological alterations were also detected. The clinical protocol of 5-FU promotes toxicity in the liver, kidneys, and lungs of healthy rats, resulting in different levels of histological and biochemical alterations. These results will be useful in the search for new adjuvants to attenuate the adverse effects of 5-FU in such organs.

## 1. Introduction

Cancer is the second leading cause of death worldwide. The incidence and mortality rates of cancer continue to rise globally, with an estimated 19.3 million new cases and 10 million deaths in 2020 [1,2]. Cancer treatment options include surgery, radiotherapy, and/or chemotherapy and different factors are involved in the effectiveness of the treatment including socioeconomic factors, diagnosis, affordable drugs, and proper protocol [3]. 5-FU is widely used to treat solid cancers, especially colorectal cancer, which has one of the highest incidences and mortality rates [4,5]. Over the years, the World Health Organization (2020) [6] has been including 5-FU in the list of the most important and necessary drugs in the basic health system, given its low cost and efficiency [7]. Additionally, the use of 5-FU has been studied in new approaches, such as 5-FU nanoparticles associated with other molecules [8,9], as well as in applications for treating other diseases, including vitiligo [10]. Thus, it is evident that the side effects of 5-FU must be elucidated in order to improve safety and treatment effectiveness.

The mechanism of action of 5-FU depends on anabolic reactions (synthesis) that generate active metabolites: fluoro-deoxyuridine monophosphate (FdUMP), fluorodeoxyuridine triphosphate (FdUTP), and fluorouridine triphosphate (FUTP) [11]. FdUMP binds to thymidylate synthase (TS) and irreversibly blocks its action, leading to the inhibition of DNA synthesis. FdUTP and FUTP are mis-incorporated into DNA and RNA, respectively, leading to the disruption of genetic material synthesis and, consequently, cell death [12,13]. 5-FU also enhances the mitochondrial production of reactive oxygen species (ROS) through a p53-dependent signaling pathway, where cytochrome C is released from mitochondria, generating oxidative damage [14].

As a chemotherapeutic agent, the action of 5-FU presents a systemic effect, i.e., healthy tissue cells are also affected by the 5-FU toxicity [15,16]. This systemic action of 5-FU is correlated to a series of side effects that can lead to treatment interruption, compromising its efficiency [17,18]. Most of the published data indicate that the side effects of 5-FU are predominant on organs containing a high level of proliferative cells, such as the intestines and bone marrow [19,20,21]. However, vital organs with a low-proliferation capacity, such as the liver, kidneys, and lungs, are constantly exposed to 5-FU metabolites and certainly can be affected negatively [22]. Side effects, such as veno-occlusive disease [23], hepatitis, steatosis, steatohepatitis [24], nephropathy [25], and chest pain [26], have been reported in patients after 5-FU treatment. These pathological processes might be induced by the oxidative stress and inflammation resulting from 5-FU chemotherapy [27,28,29,30].

To alleviate the side effects, the administration of the chemotherapeutics agent needs to be carried out with adequate dosage in periodic cycles, to allow healthy tissues to recover [31]. The 5-FU chemotherapy is performed with one cycle of drug doses applied on alternate days, in approximately two weeks. However, studies conducted with 5-FU in animal models, in most cases, use single high doses, which differ from the chemotherapy protocol applied to human cancer treatments [32]. Therefore, in this study, we investigate the effects of 5-FU clinical protocol on the liver, kidneys, and lungs of healthy rats. Our data demonstrated that 5-FU treatment resulted in histological disruption, biochemical alterations, and inflammatory responses in the analyzed organs, and pointed out that the clinical protocol of 5-FU promotes toxicity in organs with a low-proliferation capacity.

## 2. Materials and Methods

### 2.1. Animals

The animal protocol used in this study was approved by the Committee for Ethical Conduct in the Use of Animals in Experimentation of the State University of Maringá (CEUA, n° 4422140918). A total of 14 male Wistar rats (weight 270.6 ± 8.2 g) were acquired through the Central Animal Facility of the State University of Maringá. The rats were acclimated to standard laboratory conditions with a light/dark cycle of 12 h and a temperature of 22 ± 1 °C.

### 2.2. Experimental Protocol

The experiment was designed in two groups (*n* = 7): control and treated groups. The rats of the treated group received 5-FU (fluorouracil 50 mg/mL-Neugrast^®^) intraperitoneally for 4 consecutive days at a dose of 15 mg/kg (equivalent for humans), and then the dose was reduced to 6 mg/kg for 4 alternate days. The last dose (15 mg/kg) was administered on the 14th day of the treatment. Next, euthanasia was performed on the 15th day. Control rats were treated with vehicle (0.9% saline solution) and then submitted to the same protocol for the 5-FU treated rats. Euthanasia was performed with a lethal dose (120 mg/kg) of Tiopental Sodium^®^ (Cristália Pharmaceutical Chemicals LTDA, Itapira, SP, Brazil) intraperitoneally.

### 2.3. Weights and Histological Analysis of Organs

The weight of the organ was normalized according to the percentage of body weight of the rats (relative organ weight = organ weight/animal body weight × 100). The square lobe of the liver, the right kidney, and the left lung were separated for the histological techniques. The segments were immediately fixed in 4% paraformaldehyde (pH 7.4) for 24 h, dehydrated in a series of alcohol, cleared in xylol, and embedded in paraffin. The material was cut into a semiserial microtome with a thickness of 5 μm and kept on slides for staining.

Images were captured under an optical microscope with the aid of a capture system with a high-resolution camera. The morphometric measurements were performed using Image-Pro Plus-Media Cybernetics image analysis software.

#### 2.3.1. Analysis of Liver Histology

The histological sections of the liver were stained with hematoxylin-eosin (HE) or periodic acid Schiff (PAS). HE was used to evaluate the semiquantitative alterations in the morphology of the liver, and the following criteria were chosen: (A) Score of steatosis; (B) Portal infiltrate; (C) Lobular infiltrate. For each criterion scores were used for classification. Steatosis and lobular infiltrate were analyzed in 40 random fields per animal, whereas portal infiltrate was analyzed in 20 random fields per animal.

PAS staining was used for the morphometric analysis of the cytoplasmic and nuclear areas of 200 random hepatocytes. The diameter of 100 hepatic sinusoids per animal was also measured. The results were expressed in μm^2^.

#### 2.3.2. Analysis of Kidney Histology

The histological sections of the kidney were stained with HE. Measurements were made in the renal cortex based on the methods of Marcelino et al. (2020) [33]. The areas of the corpuscle and the visceral layer were measured in 30 glomeruli per animal. The area of Bowman’s space was calculated, which resulted from the measurements of the area of the corpuscle subtracted from the area of the visceral layer (corpuscle − visceral layer = Bowman’s space). These results were expressed in μm^2^.

The internal and external areas of both the proximal and distal tubules were also measured, and the thickness of each tubule was calculated by subtracting the external area from the internal area (external area − internal area = thickness). Measurements were performed in 80 distal and 80 proximal tubules per animal. The results were expressed in μm^2^.

The number of renal corpuscles in the cortex was quantified in 50 random fields per animal. The results were expressed as the number of corpuscles per mm^2^.

#### 2.3.3. Analysis of Lung Histology

Histological sections stained with HE and Picrosirius red were used to evaluate lung morphology. HE was used to evaluate edema, area, and alveolar thickness. The edema analyses were performed in 30 random fields per animal (each field with a total of 1451.031 μm^2^). The alveolar analyses were performed in 300 alveoli per animal, and the thickness measurements were obtained using the average of 5 measurements around the alveoli walls. The results were expressed in μm^2^.

We also performed a quantification of hemorrhagic foci, perivascular focal infiltrate, and diffuse infiltrate in the lung parenchyma. This quantification was performed in relation to the number of foci per area (lower magnification) of the histological tissue. The results were expressed in mm^2^ of lung tissue.

Picrosirius red was used to quantify collagen fibers of type I (orange–yellowish to orange and red birefringence), type III (green or yellow–green birefringence) and total collagen (type I + type III = total collagen). A total of 30 random fields were captured per animal in a trinocular light microscope (NIKON^®^ Eclipse 80i) with a polarization filter coupled to a high-definition digital video camera. The results were expressed as the percentage of the area containing collagen in relation to the total area.

### 2.4. Evaluation of Apoptosis (TUNEL)

The apoptosis rate was evaluated in histological sections obtained from segments of the square lobe of the liver, the right kidney, and the left lung. This evaluation was performed using the deoxynucleotidyl transferase terminal labeling assay kit. dUTP (TUNEL) staining was performed according to the manufacturer’s instructions (In Situ Cell Death Detection Kit, POD-Roche).

TUNEL is a dUTP-mediated cutting-edge labeling assay, and the reagents in the kit allow the labeling of the 3’OH terminus of the free (exposed) DNA. The nucleotides contained in the kit solution are enzymatically linked to DNA by dUTP, forming an oligomer by digoxigenin. The antidigoxigenin antibody was incubated, and antigen–antibody binding was detected using immunoperoxidase followed by DAB chromogen (3,3′-diaminobenzidine). For each tissue under study, the assay was standardized using the positive control of apoptosis (TUNEL-positive nuclei treated with DNase I) and the negative control of apoptosis (TUNEL-negative nuclei without dUTP). The slides obtained after the test were counterstained with hematoxylin, and the images were captured under an optical microscope with the aid of a capture system with a high-resolution camera.

Ten fields were randomly selected from the liver, kidney, and lung slides of each experimental animal. The number of cells/nuclei positive or negative for TUNEL was defined in comparison to the standardized positive and negative control slides. The number of cells positive for TUNEL was quantified as the percentage of total cells per animal.

### 2.5. Oxidative Stress Markers

For oxidative stress analysis, the major lobe of the liver, the left kidney, and the right lung were separated. The segments were weighed and homogenized in potassium phosphate buffer (200 mM) pH 6.5 with a volume of 10× the weight of the sample for the kidney and liver and 7× for the lung. Part of this homogenate was separated for the reduced glutathione (GSH) evaluation. The remainder was centrifuged at 9000 rpm for 20 min, resulting in a supernatant that was aliquoted separately for catalase (CAT), superoxide dismutase (SOD), glutathione-S-transferase (GST), and lipid hydroperoxides (LOOH) measurements.

#### 2.5.1. Reduced Glutathione (GSH) Measurements

A total of 48 µL of trichloroacetic acid (TCA) was added to the homogenate of the samples for protein precipitation and then centrifuged. After centrifugation the supernatant was placed on plates, and a solution of Tris-HCl (0.4 M) pH 8.9 and DTNB (5.5′-dithiobis 2-nitrobenzoic acid, 10 mM) was added [34]. The GSH level was measured spectrophotometrically at 415 nm. The values were plotted on a standard GSH curve and expressed as μg GSH/g tissue.

#### 2.5.2. Enzymatic Activity of Glutathione-S-Transferase (GST)

The supernatant of each sample was diluted in potassium phosphate buffer (0.2 M, pH: 6.5) and CDNB (1-chloro-2,4-dinitrobenzene) and GSH solutions were added [34]. The GST level was measured at 340 nm with an extinction coefficient of 9.6 mM cm^−1^. The results were expressed as μmol/min/mg of protein.

#### 2.5.3. Catalase (CAT) Enzymatic Activity

The method used was based on Aebi (1984) [35]. The supernatant was diluted in a potassium phosphate buffer (0.2 M, pH 6.5). The reaction was performed by adding Tris-HCl + 0.1 M EDTA buffer (pH 8.5) and H_2_O_2._ CAT activity was measured spectrophotometrically at 240 nm. The results were expressed as μmol/min/mg of protein.

#### 2.5.4. Enzymatic Activity of Superoxide Dismutase (SOD)

SOD activity was determined by the ability of this enzyme to inhibit the autoxidation of pyrogallol. The reaction was performed using the supernatant with the addition of Tris-HCl EDTA buffer (pH = 7.5) and pyrogallol [34]. The solution was incubated at room temperature for 20 min, and then 1 M HCl was added to stop the reaction. Finally, the enzymatic activity of SOD was measured at 405 nm. The results were expressed in U of SOD/mg of protein.

#### 2.5.5. Levels of Lipid Hydroperoxides (LOOH)

The supernatant of each sample was diluted in 30% methanol. Subsequently, a solution containing 90% methanol xylenol orange, sulfuric acid (H_2_SO_4_, 25 mM), butylated hydroxytoluene (BHT, 4 mM), and FeSO_4_NH_4_ (250 mM) was added to the reaction [34]. The reaction was incubated for 30 min in the dark and, after this period, the absorbance was measured at 560 nm. LOOH concentrations were determined using a molar extinction coefficient of 4.3 mM cm^-1^, and the results were expressed as mmol/mg of tissue.

#### 2.5.6. Total Protein Concentration

The total protein concentration was obtained using the commercial Pierce™ BCA Protein Assay kit, following the manufacturer’s recommendations. The absorbance was measured at 562 nm and the results were expressed in µg/mL.

### 2.6. Analysis of Inflammatory Process Markers

Segments of the major lobe of the liver, the left kidney, and the right lung were separated for the inflammatory analyses. The precipitate from the tissue homogenate was resuspended in potassium phosphate buffer solution (0.08 M) with hexadecyltrimethylammonium (HTAB) (pH 5.4). The samples were homogenized and centrifuged for 20 min at 11,000 rpm, 4 °C. The supernatant was used to analyze the activity of the myeloperoxidase (MPO) and N-acetyl-glucosaminidase (NAG) enzymes.

#### 2.6.1. Myeloperoxidase (MPO) Enzymatic Activity

The MPO activity was analyzed after the addition of hydrogen peroxide (H_2_O_2_) and tetramethylbenzidine (TMB) to the supernatant obtained from the samples. The absorbance was measured at 620 nm and the results were expressed in the optical density unit mDO/mg of protein. 

#### 2.6.2. Activity of the Enzyme N-Acetyl-Glucosaminidase (NAG)

Citrate buffer (50 mM, pH 4.5) and NAG solution (2.24 mM) were added to the supernatant for the analysis of NAG. The solution was incubated for 60 min at 37 °C, and then the reaction was stopped with the addition of a glycine buffer (200 mM, pH 10.4). The absorbance was measured at 405 nm and the results were expressed in mDO/mg of protein.

#### 2.6.3. Quantification of Nitric Oxide (NO)

The Griess reaction was used to indirectly evaluate NO from its by-product, nitrite [36]. Specific segments of each tissue were shredded in 0.1 M PBS (pH 7.4) and then centrifuged. Phosphoric acid, sulfanilamide, distilled water, and N-1-naphthylenediamide (NED) were added into the supernatant samples to compete with the nitrite. The product of this reaction was measured at 540 nm. The nitrite concentrations were calculated using a standard curve ranging from 100 to 1.56 μM of sodium nitrite (NaNO_2_). The nitrite levels were expressed as μM. 

#### 2.6.4. Measurements of Interleukin Levels

The tissue concentration of the interleukin 1β (IL-1β), interleukin 6 (IL-6), and interleukin 10 (IL-10) were quantified from homogenates. Interleukin analyses were performed using enzyme-linked immunosorbent assay (ELISA) kits (R&D Systems) according to the manufacturer’s protocol. The absorbance was measured at 450 nm. Interleukin concentrations were expressed as pg/mL.

### 2.7. Functional Analyses of Liver and Kidney

Blood was collected via cardiac puncture and the plasma was used to evaluate aspartate aminotransferase (AST), alanine aminotransferase (ALT), urea, creatinine, and uric acid. The analyses were performed using the commercial kit Analisa^®^, and each reading followed the manufacturer’s recommendations.

### 2.8. Statistical Analysis

Statistical analyses were performed using GraphPad Prism 7 software. The normality of the results was analyzed using the Shapiro–Wilk test. For the parametric data, the comparison between the groups was performed using the Student’s t test. For nonparametric data, the comparison between groups was performed using the Mann–Whitney test. For such analyses, *p* values less than 0.05 were considered statistically significant. The results are expressed as the mean ± standard error (parametric) or the median ± lower limit-upper limit (nonparametric).

## 3. Results

### 3.1. Treatment with 5-FU Promotes Morphological and Morphometric Alterations in the Liver, Kidney, and Lung

**Figure 1:**
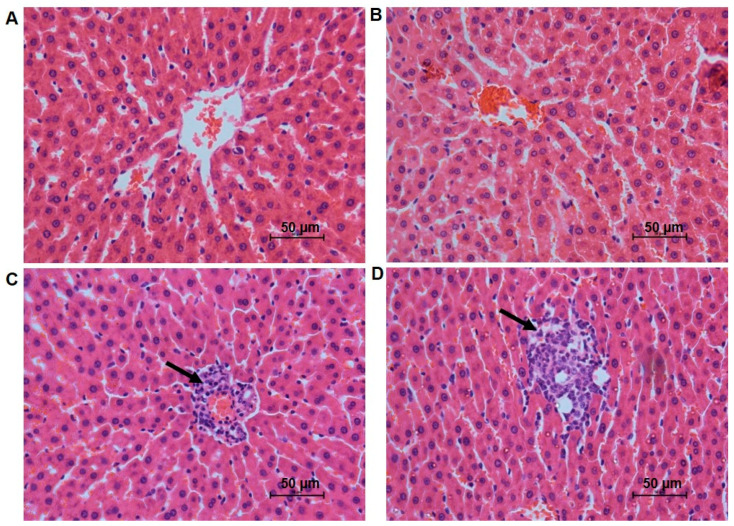
Photomicrographs of the liver tissue of rats in the studied groups: (**A**) Centrilobular region without steatosis, control group; (**B**) Centrilobular region with steatosis, group treated with the 5-FU clinical protocol; (**C**) Portal region with inflammatory cell infiltrate, group treated with the 5-FU clinical protocol; (**D**) Centrilobular region with inflammatory cell infiltrate, group treated with the 5-FU clinical protocol. Focus of inflammatory cells (arrow). HE staining, 40×.

To investigate the impact of 5-FU on the architecture of the liver, kidney, and lung, a set of morphological and morphometric parameters were analyzed (Table 1 and Table 2). The 5-FU treatment did not change the relative weight of the liver. However, the hepatocytes surface area and the nuclear area and diameter of the sinusoidal capillaries increased significantly in the 5-FU group when compared to the control group (Table 1). The liver of the animals treated with 5-FU also presented steatosis in moderate (28% of the animals) and severe (71.4% of the animals) rates, while the control group presented basal rates of steatosis (Figure 1B, Table 2). Approximately 15% of the 5-FU treated rats were classified with a severe portal inflammatory infiltrate (Figure 1C), 70% with a moderate, and 15% with a mild portal inflammatory infiltrate. In the control group, 28% of the animals did not present portal inflammatory infiltrate, ~43% presented mild and 28% moderate rates of portal infiltrate (Table 2). Lobular inflammation was predominantly classified between mild and moderate in the 5-FU treated group (Figure 1D), whereas the control group presented mild or no inflammatory lobular infiltrate (Table 2). 

**Figure 2:**
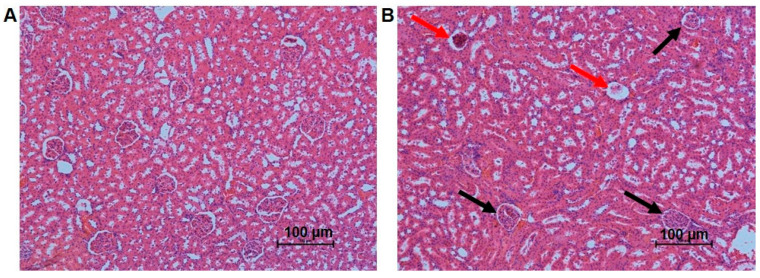
Photomicrographs of the renal tissue of rats in the studied groups: (**A**) control group; (**B**) group treated with the 5-FU clinical protocol. Damaged renal corpuscles (red arrows). Reduced number of renal corpuscles (black arrows). HE staining, 20×

The analyses of the kidney revealed that the treatment with 5-FU did not significantly alter the relative weight of this organ (Table 1). However, some morphometric parameters have changed, including the renal corpuscle and distal tubules. There was a significant increase in renal corpuscle parameters (corpuscle area, visceral layer area, and Bowman space) in rats treated with 5-FU. The density of renal corpuscles per mm^2^ decreased significantly in the 5-FU group compared with the control. Clinical treatment with 5-FU did not significantly alter the parameters of the proximal tubules (PT); although, it increased the external area, internal area, and the thickness of the distal tubules (DT) when compared to the control group (Table 1, Figure 2A,B).

**Figure 3:**
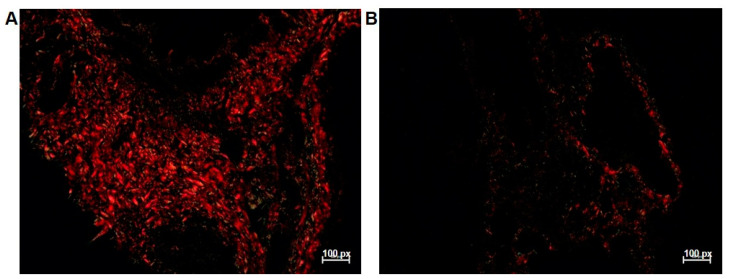
Photomicrographs of the deposition of collagen fibers type I (orange–yellowish to orange and red birefringence) and type III (green or yellow–green birefringence) of the lung tissue of rats in the studied groups: (**A**) control group; (**B**) group treated with the 5-FU clinical protocol. Picrosirius-red staining, 20× (with polarization).

**Figure 4:**
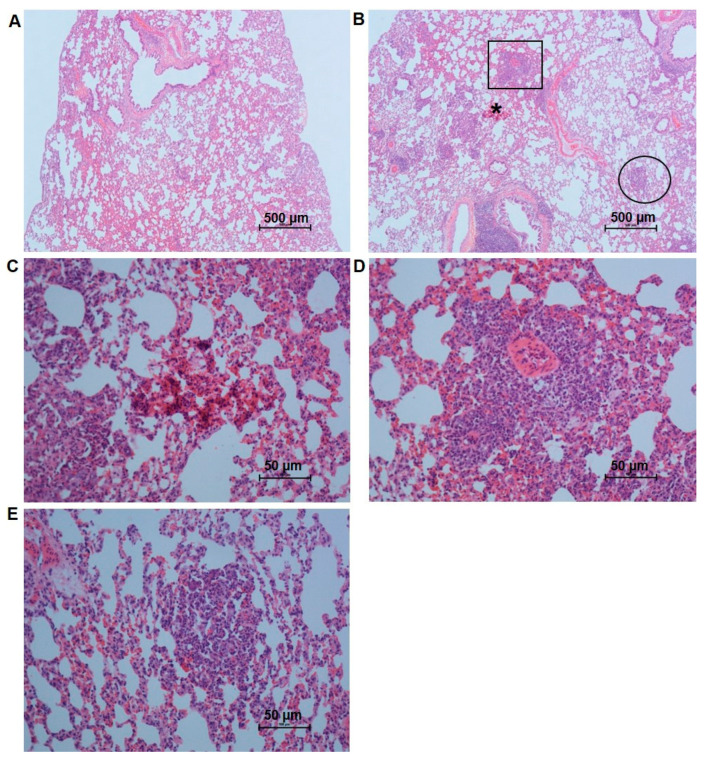
Photomicrographs of lung tissue sections of rats from the different groups, obtained with a 4×objective and stained with HE: (**A**) Control group, showing the normal architecture of the tissue; (**B**) 5-FU group showing perivascular focal inflammatory infiltrate (square), diffuse infiltrate in the lung parenchyma (circle), and hemorrhagic focus (*); (**C**) Hemorrhagic focus in the treated group at 40× magnification; (**D**) Perivascular focal inflammatory infiltrate at 40× magnification; (**E**) Diffuse infiltrates in the lung parenchyma in the treated group at 40× magnification.

**Figure 5:**
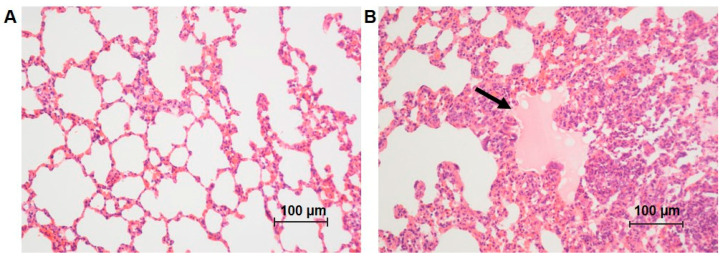
Photomicrographs of lung tissue sections of rats from the different groups: (**A**) control group, showing the normal architecture of the tissue; (**B**) 5-FU group showing areas of edema (arrow) and increase in the thickness of the alveolar septum. HE staining, 20×.

Lung weight was significantly higher in the 5-FU-treated group. (Table 1). The quantification of type I, III, and total collagen (Figure 3A,B) was significantly lower in rats treated with 5-FU. However, the number of hemorrhagic foci per mm^2^ of lung tissue (Figure 4B,C), the presence of perivascular focal infiltrates (Figure 4B–D), diffuse infiltrates in the lung parenchyma (Figure 4B–E), and edema were increased in the 5-FU group (Table 1, Figure 5A,B). Additionally, there was a significant increase in the thickness of the alveolar septum and a reduction in the alveolar area of the animals treated with 5-FU (Table 1, Figure 5A,B).

### 3.2. 5-FU Treatment Increases Cell Death in the Liver

**Figure 6:**
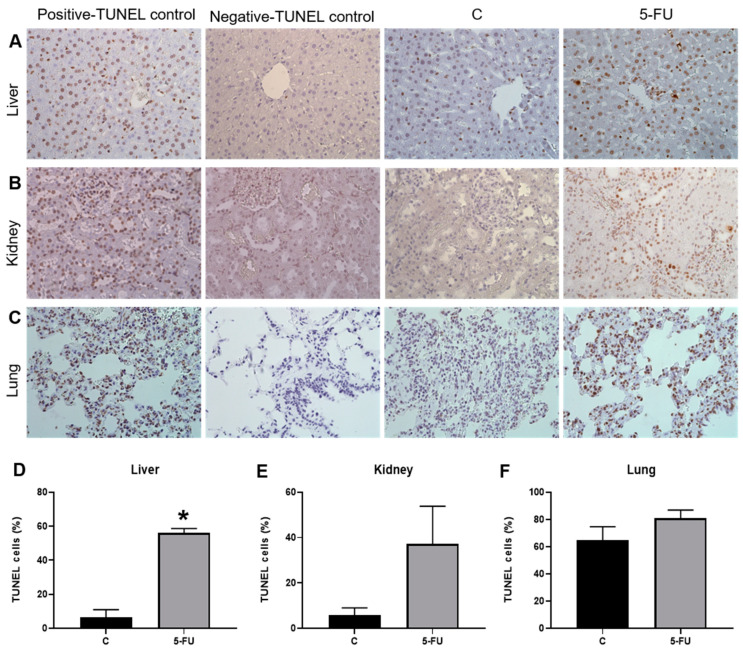
Photomicrographs of tissue sections of rats from the different groups, obtained with a 40× objective and TUNEL assay: (**A**) Liver; (**B**) Kidney; (**C**) Lung. Percentage of TUNEL-positive nuclei in the tissues of rats from the different groups: (**D**) Liver; (**E**) Kidney; (**F**) Lung. *, significant difference (*p* < 0.05) compared to the control group. Data are expressed as the mean ± standard error (para-metric), *n* = 4. C—control group; 5-FU—treated group.

To evaluate the impact of 5-FU treatment in the cell death by apoptosis, TUNEL reaction was performed (Figure 6). In the liver, 5-FU treatment resulted in an increase of the apoptosis (Figure 6A–D). TUNEL-positive cells/nuclei quantified in the kidneys (Figure 6B–E) and lungs (Figure 6C–F) showed no significant differences in relation to the control and 5-FU-treated groups.

### 3.3. 5-FU Treatment Modulates Oxidative Stress Markers

The measurements of the oxidative parameters in the liver showed that treatment with 5-FU induced a significant reduction in the activity of CAT and GST enzymes and in the levels of GSH; however, the activity of SOD was not changed between the groups (Table 3). Moreover, the levels of LOOH increased after 5-FU treatment (Table 3).

The analysis of the kidney tissue showed a significant increase in the enzymatic activity of CAT and SOD in the treated group. The levels of GSH and LOOH were significantly reduced in the kidney tissue of 5-FU treated rats, whereas the GST activity was not impaired by the treatment (Table 3).

In the lung tissue the activity of SOD, CAT, and GST and the levels of GSH decreased significantly in the 5-FU group. The analysis of the LOOH levels showed an increase in this oxidative stress marker in the lung tissue of treated rats compared to the control group. There was also a significant reduction in the concentration of total proteins obtained from the lung in the treated group (Table 3).

### 3.4. Inflammatory Response Was Modulated by 5-FU Treatment

In the liver tissue, the activity of MPO and NAG and the levels of NO and IL-1β increased significantly after 5-FU treatment. The concentration of IL-6 was significantly lower in the 5-FU-treated group and there was no significant difference between the groups in the quantification of IL-10 (Table 4). In the renal tissue, the enzymatic activity of MPO and NAG decreased significantly in the 5-FU-treated group. There were no statistically significant differences in the levels of NO, IL-1β, IL-6, and IL-10 in the 5-FU-treated group in relation to the control (Table 4). In the lung tissue, the enzymatic activity of NAG and MPO and the NO levels increased significantly in the 5-FU group, whereas the IL-6 concentration was negatively regulated by the treatment (Table 4).

### 3.5. 5-FU Treatment Impacts on the Function of the Liver and Kidneys

**Figure 7:**
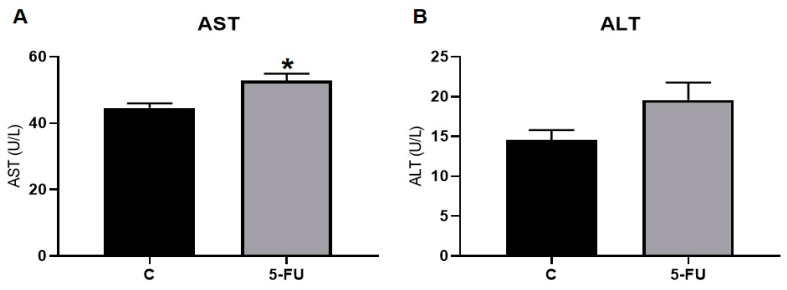
Liver function markers of rats treated with the 5-FU clinical protocol: (**A**) Serum aspartate aminotransferase (AST) enzymatic activity; (**B**) Serum alanine aminotransferase (ALT). *, significant difference (*p* < 0.05) compared to the control group. Data are expressed as the mean ± standard error (parametric), *n* = 7.

**Figure 8:**
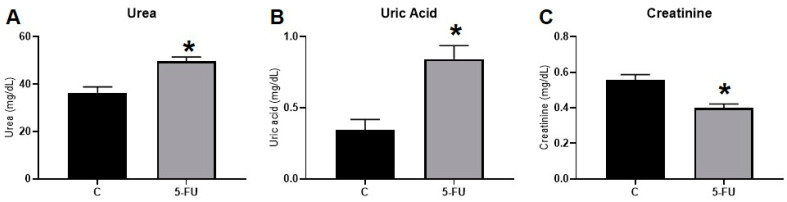
Markers of renal function in rats treated with the 5-FU clinical protocol: (**A**) Levels of urea; (**B**) Uric acid (**C**); Serum creatinine. *, significant difference (*p* < 0.05) compared to the control group. Data are expressed as the mean ± standard error (parametric) and median ± lower limit-upper limit (nonparametric), *n* = 7.

The functional analysis revealed that the serum AST values increased significantly after 5-FU treatment; however, there was no difference in the serum ALT values between the groups (Figure 7A,B). Moreover, the administration of 5-FU promoted a significant increase in the serum urea and uric acid values and decreased creatinine levels in rats (Figure 8A–C).

## 4. Discussion

Most studies conducted with 5-FU use doses higher than the dose used in clinical practice [30,37,38,39]. Therefore, little is known about the effects of this drug on vital organs such as the liver, kidneys, and lungs after the treatment suggested by the manufacturer. In this study, we evaluated the effects of the suggested clinical protocol of 5-FU chemotherapy on these three organs of healthy Wistar rats. For the first time, we demonstrated that 5-FUtreatment, following the clinical protocol, promotes histological, biochemical, oxidative stress, and inflammatory modulations in the evaluated organs.

In this study, the treatment with 5-FU was performed intraperitoneally [37,38,40]. Although 5-FU is administered intravenously in humans, some studies have shown that the effectiveness of 5-FU was not affected by the route of administration [40,41]. After administration, most of the 5-FU is immediately catabolized by the dihydropyrimidine dehydrogenase (DPD) enzyme. DPD is responsible for catalyzing the conversion reaction of 5-FU into dihydrofluorouracil (DHFU), which is then catabolized into 5-fluoro-5,6-dihydrouracil (5-FUH2) and then into fluoro-beta-ureidopropionate (FUPA) [42]. DPD is expressed in large quantities in the liver, so this organ is mainly responsible for the degradation reactions of 5-FU. In the kidneys, FUPA is transformed into fluoro-beta-alanine (FBAL), which is excreted [12]. FBAL is a 5-FU inactive metabolite; however, unlike the others, this metabolite is often associated with renal dysfunction [43,44]. The detailed way in which FBAL affects the kidneys is still not well understood. The lungs are also reported to be a site of 5-FU elimination [22,45]. Under normal conditions, only a small percentage of the 5-FU dose undergoes anabolic reactions generating active metabolites (nucleotide anabolites) with cytotoxic effects [46]. Although catabolism is predominant, 5-FU anabolism occurs in all tissues; i.e., its action is systemic [22].

We demonstrated that the passage of 5-FU through the liver led to functional damage, accessed by elevated serum AST activity (Figure 7). According to Ozer et al. (2008) [47], AST is considered one of the markers of liver function and serum elevation of this marker can be associated with liver toxicity. This study also demonstrated that 5-FU promoted oxidative stress in the liver tissue. Our data pointed to a reduction in the activity of the CAT and GST enzymes and of the antioxidant molecule GSH on the liver of treated animals. In addition, an increase in LOOH was observed. The increase in LOOH characterizes lipid peroxidation caused by a redox imbalance, and is related to cell damage in this organ [48,49]. 

5-FU is known to generate reactive species of mitochondrial oxygen through a p53-dependent signaling pathway, which culminates in the release of cytochrome C from mitochondria [49,50]. This event causes an electron deviation from the transport system towards oxygen, resulting in the formation of superoxide radicals (O_2_^•−^), a type of reactive oxygen species [51]. O_2_^•−^ is highly harmful to cells because it can be converted into H_2_O_2_ by the Fenton reaction and then transformed into a hydroxyl radical (OH^•^) that reacts with lipids, causing lipid peroxidation and the consequent formation of LOOH [52]. Lesions related to lipid peroxidation and oxidative stress pathways can cause hepatic steatosis, an accumulation of triglycerides within hepatocytes [53]. This pathology is often associated with the administration of 5-FU [53,54]. In a study carried out with 30 patients with metastatic colorectal carcinoma it was observed that about 30% of the patients had hepatic steatosis after 5-FU treatment [55]. According to Sørensen et al. (1995) [55], tissue injury caused by chemotherapy recruits cytokines, including tumor necrosis factor IL-1, and IFN-a, resulting in increased hepatic fatty acid synthesis. Corroborating this idea, in our study we observed an increase in the degree of steatosis in the liver of the 5-FU-treated rats when compared to the controls (Figure 1).

Studies have shown that the presence of oxidative stress and steatosis makes the liver more susceptible to inflammatory reactions [56,57]. This was confirmed in our results after we observed an increase in the activity of the inflammatory process enzyme markers (MPO and NAG) and NO in the liver of treated rats (Table 4). We also observed an increased concentration of IL-1β in the liver of rats treated with 5-FU (Table 4); however, IL-6 concentration decreased in the liver. Generally, an increase in IL-6 was observed with the use of 5-FU [58], but its reduction has also been observed in the plasma of patients treated with this drug [59]. IL-1 is produced at the onset of inflammation and then stimulates the production of IL-6 [58]. According to Matthews et al. (2010) [60], IL-6 deficiency may be a consequence of mitochondrial dysfunction. 5-FU is often associated with reduced membrane potential and mitochondrial collapse [61]. Another fact that indicates the inflammatory process in this case is the increase in inflammatory foci in the portal and lobular areas of the liver tissue from 5-FU-treated rats (Figure 1). Some authors claim that in cases of chronic inflammation the presence of inflammatory infiltrates in the liver portal area overlaps with that of lobular region [62,63]; this data corroborates our findings. A study performed by Sommer et al. (2017) [30], in which high doses (200 mg/kg) of 5-FU were used, also showed oxidative damage and inflammation in the liver of mice after 24 h of treatment.

Oxidative stress and inflammation may be responsible for histological changes in the liver tissue, such as the dilation of sinusoid capillaries [64]. A significant increase in the diameter of the sinusoid capillaries of rats treated with 5-FU was observed in this study. Another histological alteration was the increased nuclei and superficial areas of the hepatocytes after 5-FU treatment (Table 1). This increase in the nuclei and superficial area indicates hepatocyte hypertrophy. According to Neufeld and Edgar (1998) [65], cell growth rate is associated with its rate of division. Therefore, cell hypertrophy may be related to the cell cycle block [65], which is a consequence of the action of 5-FU [13]. Moreover, the oxidative stress damage and cell cycle blockage mediated by 5-FU can induce apoptosis cascade activation and cell death [66,67]. This was confirmed when we observed a significant increase in apoptotic cells (TUNEL-positive) in the liver of rats treated with 5-FU compared with the controls (Figure 6).

In the present study we observed that the passage of 5-FU metabolites in the kidneys did not promote oxidative stress damage or inflammation (Table 3 and Table 4). Our results show that there was no lipid peroxidation in the kidneys due to increased SOD and CAT activity, and GSH intake. One way to avoid lipid peroxidation by an excess of ROS is to increase the activity of endogenous antioxidants, maintaining a redox balance [40]. SOD performs the dismutation of O_2_^−^ to H_2_O_2,_ and CAT converts this compound into H_2_O and O_2_ or is eliminated by glutathione peroxidase using GSH via GST catalysis [68]. We observed that the activities of the MPO and NAG enzymes significantly decreased in the kidneys of the 5-FU treated group, whereas the interleukins (IL-1β, IL-6 and IL-10) showed no significant difference; i.e., there was no inflammation in this organ due to 5-FU treatment. However, Liu et al. (2018) [69] observed an increase in MPO, neutrophils, and macrophages in the kidneys when a higher dose of 5-FU (200 mg/kg) was administered for 4 days and euthanasia was performed on the 18th day. In addition, increased expressions of proinflammatory cytokines, such as IL-1β, IL-6, and TNF-α, were found [69]. Such differences may be related to the higher dose of 5-FU used by Liu et al. in the animal’s treatment, which differs from that used in our protocol and in the clinical practice [70]. According to Matsushita et al. (2020) [71], kidneys are compensatory organs and, even though they are a major target of toxicity by xenobiotic agents, they can maintain some homeostasis.

Even without inflammation or oxidative stress, alterations were observed in the histological analysis of the kidneys. There was a significant increase in the inner and outer areas and thickness of the DT in the 5-FU group compared to the control (Table 1). The reason for tubule hypertrophy was related to the increased activity of ornithine decarboxylase (ODC), a rate-limiting enzyme in polyamine biosynthesis [72]. According to Koseki et al. (2016) [73], in a study with cancer stem cells, 5-FU reduced polyamine levels; however, it was observed that in noncancerous cells polyamine levels were elevated in the presence of 5-FU. This result indicates that the DT hypertrophy may be related to the increased ODC activity, as the cells in our study are not cancerous. However, the ODC activity was not measured in our study to confirm this hypothesis. Regarding glomerular parameters, increases in the corpuscle, visceral layer, and Bowman space areas were observed in the glomeruli of the treated group. According to Tobar et al. (2013) [74], the increase in the glomerular space (Bowman’s space) is associated with glomerular hyperfiltration, which results in increased hydrostatic pressure in this space and its consequent dilation. This glomerular hyperfiltration may be the result of the loss of functional glomeruli and the increased compensatory filtration on the remaining ones [75,76]. The chemotherapy is responsible for damage to the glomeruli and consequent reduction in glomerular density [77]. Our data support this idea, as we observed a significant reduction in the density of glomerular corpuscles per area in the group treated with 5-FU (Figure 2).

When a significant number of functional glomeruli are lost, kidney function can decline [75]. Therefore, the analysis of renal function showed that plasma substances (urea, uric acid, and creatinine), which serve as functional markers of the kidneys, underwent alterations. Such markers are constantly excreted by the kidneys; therefore, in conditions of injury, an increase in serum levels can be observed [78]. In this study, we observed a significant increase in urea and uric acid in the plasma of animals treated with 5-FU (Figure 8). Creatinine, in this case, showed a significant reduction in treated rats compared to untreated ones. Creatinine is a product of creatine metabolism, which is largely produced in the liver and stored in skeletal muscle to be used as an energy source [79,80]. In the plasma, creatinine concentration can be significantly affected by causes other than renal dysfunction, such as by nutritional status and, mainly, by liver diseases [79,81]. According to Slack et al. (2010) [79], a reduction in serum creatinine is observed in patients with chronic liver disease, and this happens because there is a decrease of up to 50% in the hepatic production of creatine. In our study, we saw that the liver was seriously affected by the treatment, which could explain the reduction found in serum creatinine. To better understand the alterations found in the renal tissue, we evaluated cell death using the TUNEL analysis. However, TUNEL staining showed no significant difference between the renal tissue from treated and control groups. Moore et al. (2021) [82] show that up to 10% of TUNEL-positive cells are considered the normal baseline for untreated kidney tissue samples, as the kidneys are among the organs that produce large amounts of DNAse I. DNAse I is normally secreted by tubular epithelial cells to destroy viruses and bacteria in the urine [82]. The absence of differences between the analyzed groups may be related to this organ specificity.

Treatment with 5-FU resulted in lung oxidative stress, inflammation, and histopathological lesions. Naturally, the lung is the organ that is most susceptible to oxidative stress because it is directly exposed to the highest oxygen tensions [83]. Therefore, a greater protection mechanism is expected to function in this tissue [84]. Nevertheless, the lungs, after treatment with 5-FU, presented a reduction in endogenous antioxidant defenses (activity of the enzymes SOD, CAT, and GST and GSH levels), which may have contributed to the increased LOOH levels resulting from the oxidative stress (Table 3). As mentioned earlier, 5-FU generates mitochondrial ROS [14]. However, although mitochondrial ROS are generally considered toxic, they can also have beneficial effects, such as inducing mitophagy, the selective removal of damaged mitochondria [85]. Larson-Casey et al. (2016) [86] showed that mitophagy contributes to the resistance to apoptosis of alveolar macrophages. This fact may support our TUNEL results that indicate the maintenance of the apoptosis rates, despite the oxidative stress. 

The lung tissue also showed inflammation because of the MPO and NAG enzyme activity and the increased NO levels, despite the reduction in the proinflammatory cytokine IL-6, as was observed in the liver. We had limitations in the analysis of the cytokines IL-1β and IL-10 in the lung tissue due to a lack of material. However, the analyses of the other parameters support the idea that inflammation occurred in this organ. In addition, the histological analyses of the lungs of treated rats showed the presence of hemorrhagic foci and inflammatory infiltrates (perivascular and in the parenchyma) (Figure 4). Lung biopsies obtained by video-assisted thoracoscopic resection in patients treated with 5-FU combined with oxaliplatin showed extensive granulomatous inflammation and no evidence of fibrosis [87]. According to the literature, oxidative stress products usually activate the inflammatory process of the upper airways through the release of inflammatory mediators and cytokines [84,88]. Fernandez et al. (2018) [89], as in our study, showed that the use of 5-FU in patients with colorectal cancer led to alveolar hemorrhage.

Organ weights in toxicity analyses are usually a very important parameter [90]. Lung weight is usually calculated in proportion to pulmonary edema [91]. In this study, the relative lung weight increased significantly after the use of 5-FU, which may be an effect of the significant increase in pulmonary areas with edema (Figure 5). According to researchers, oxidative stress and the inflammatory process alter respiratory function, which results in pulmonary edema and the accumulation of inflammatory exudate [91,92]. In cases of lung injury, it is common to observe fibrosis (healing after injury), indicated by the abnormal accumulation of collagen in the extracellular tissue matrix [93]. However, in this study, the amount of type I, III, and total collagen was reduced in the treated rats (Figure 3). 5-FU metabolites are responsible for affecting protein synthesis as they are incorporated into nucleic acids, impairing the transcription process [94]. Collagen is a protein synthesized by fibroblasts, and some studies have already shown the action of 5-FU as an antiproliferative agent of fibroblasts [95], reducing the synthesis of fibronectin and collagen types I and III [94,96], similarly to our findings.

Thus, despite the antitumor effects of 5-FU, this study reveals that the clinical use of this chemotherapeutic agent is correlated with toxicity at cellular and biochemical parameters in the three organs studied. The kidneys were less injured compared to the liver and lungs but still showed significant damage. In the human body, organs and tissues work together and are highly connected through complex interactions [97]. In this study, the liver and lungs were similarly impaired. According to Kuan et al. (1998) [22], when 5-FU enters the hepatic vein, the liver and lungs start to act together due to their anatomical relationships as lungs process all the cardiac output (blood supply) that comes from the hepatic vein [22]. On the other hand, it is important to note that 5-FU has a range of action mechanisms, so cells of different origins may present different responses to this drug [98].

## 5. Conclusions

Our data depicted the histological and biochemical alterations resulting from treatment with 5-FU in the liver, kidneys, and lungs of healthy rats submitted to the clinical protocol of 5-FU. 5-FU produces histological changes in the three organs analyzed, causes physiological alterations in the liver and kidneys, and promotes oxidative stress and inflammation in the liver and lungs. Therefore, finding the potential side effects of 5-FU treatment is essential to ameliorate the harmful effects of this treatment in the whole body. It should be noted that a limitation of this study is the sample size. Larger investigations are needed to determine the mechanisms underlying the alterations found in each organ examined in this study, including preclinical studies to determine causality. When the side effects are not adequately elucidated measures to avoid these effects are no longer applied, compromising the patient’s quality of life. Thus, the detected alterations in this study can be useful in the search for new adjuvants to attenuate the adverse effects of 5-FU in liver, kidneys, and lungs.

## Figures and Tables

**Table 1:** Analyses of the relative weight and histology of the liver, kidneys, and lungs of the control and 5-FU treated groups.

	Analysis	C	5-FU	*p* Value
Liver	Liver relative weight (%)	3.803 ± 0.1355	4.241 ± 0.1904	0.0851
	Sinusoidal diameter (μm^2^)	5.562 ± 0.0734	6.022 ± 0.0975	0.0027 *
	Core area (μm^2^)	34.74 ± 0.8656	38.21 ± 0.7748	0.0113 *
	Hepatocyte area (μm^2^)	224.2 ± 4.641	261.4 ± 3.727	<0.0001 *
Kidney	Relative kidney weight (%)	0.8786 ± 0.0331	0.9214 ± 0.0191	0.2834
	Area of the corpuscle (μm^2^)	5674 ± 179.2	6959 ± 232.8	0.0009 *
	Area of the visceral layer (μm^2^)	4302 ± 173.7	5052 ± 179.7	0.0111 *
	Bowman space (μm^2^)	1372 ± 54.9	1907 ± 98.12	0.0005 *
	Number of corpuscles (mm^2^)	126 ± 1.976	107.7 ± 2.032	<0.0001 *
	External area PT (μm^2^)	13.15 ± 0.3372	14.3 ± 0.5622	0.1036
	Internal area PT (μm^2^)	3.229 ± 0.121	3.428 ± 0.2261	0.4524
	PT thickness (μm^2^)	9.92 ± 0.2286	10.88 ± 0.3859	0.0545
	External area DT (μm^2^)	9.40 ± 8.405–9.524	10.31 ± 9.783–10.84	0.0006 *
	Internal area DT (μm^2^)	3.102 ± 0.1058	3.456 ± 0.1027	0.0334 *
	DT thickness (μm^2^)	5.862 ± 0.1464	6.857 ± 0.1495	0.0005 *
Lung	Relative lung weight (%)	0.57 ± 0.0377	0.8043 ± 0.0277	0.0003 *
	Collagen type I (%/area)	28,616 ± 2325	13,718 ± 813.2	<0.0001 *
	Collagen type III (%/area)	9677 ± 1115	3872 ± 300.4	0.0003 *
	Total collagen (%/area)	37,813 ± 2566	18,175 ± 906.1	<0.0001 *
	Hemorrhagic focus (mm^2^)	0 ± 0	39.99 ± 13.55	0.0121 *
	Perivascular focal infiltrate (mm^2^)	202.1 ± 77.22	1342 ± 383.1	0.0129 *
	Diffuse infiltrate in the parenchyma (mm^2^)	13.57 ± −6.18–33.33	82.07± 26.25–137.9	0.0105 *
	Edema (μm^2^)	305.4 ± 35.38	706 ± 154.2	0.0263 *
	Alveolar area (μm^2^)	719.2 ± 50.19	531.4 ± 36.32	0.0104 *
	Thickness of the alveolar septum (μm^2^)	10 ± 0.4817	12.54 ± 0.402	0.0016 *

C—control group; 5-FU—treated group; PT—proximal tubules; DT—distal tubules. Data are expressed as the mean ± standard error (parametric) and median ± lower limit-upper limit (nonparametric), *n* = 7. * *p* < 0.05.

**Table 2:** Semiquantitative evaluation of the rat’s liver form control and 5-FU treated groups.

Description		Score	Results (*n* = 7)	
			C	5-FU
Steatosis	0–33% of the lobes	1 (mild)	100%	0%
	34–66% of the lobes	2 (moderate)	0%	28.6%
	>66% of the lobes	3 (severe)	0%	71.4%
Inflammatory portal infiltrate	Absent	0	28.6%	0%
	Focus on 1/3 of the portal tracts	1 (mild)	42.9%	14.2%
	Focus on > 1/3 and < 2/3 of portal tracts	2 (moderate)	28.5%	71.4%
	Focus on ≥ 2/3 of portal tracts	3 (severe)	0%	14.3%
Inflammatory lobular infiltrate	Absent	0	14.3%	0%
	<2 foci per field	1 (mild)	85.7%	57.1%
	2–4 foci per field	2 (moderate)	0%	42.9%
	>4 foci per field	3 (severe)	0%	0%

C—control group; 5-FU—treated group.

**Table 3:** Oxidative stress markers in the kidneys, liver, and lungs of the different groups.

	Analysis	C	5-FU	*p* Value
Liver	CAT (μmol/min/mg protein)	0.054 ± 0.0059	0.0301 ± 0.0022	0.0025 *
	SOD (U SOD/mg protein)	1.032 ± 0.0724	0.8616 ± 0.0452	0.0696
	GST (μmol/min/mg protein)	0.0584 ± 0.0026	0.0501 ± 0.002	0.0267 *
	GSH (μg GSH/g tissue)	738.8 ± 646.5–956.4	428.3 ± 291–479.6	0.0006 *
	LOOH (mmol/mg tissue)	29.77 ± 27.23–31.7	32.98 ± 29.43–42.65	0.0262 *
Kidney	CAT (μmol/min/mg protein)	0.0543 ± 0.0136	0.1413 ± 0.0142	0.0008 *
	SOD (U SOD/mg protein)	0.7785 ± 0.0629	1.138 ± 0.0468	0.0006 *
	GST (μmol/min/mg protein)	0.0315 ± 0.0034	0.0406 ± 0.0027	0.0558
	GSH (μg GSH/g tissue)	550.5 ± 32.4	388.1 ± 20.39	0.0011 *
	LOOH (mmol/mg tissue)	66.5 ± 1.646	58.59 ± 1.779	0.0068 *
Lung	CAT (μmol/min/mg protein)	0.024 ± 0.0043	0.012 ± 0.0009	0.0180 *
	SOD (U SOD/mg protein)	1.773 ± 0.1024	1.388 ± 0.0781	0.0112 *
	GST (μmol/min/mg protein)	0.0098 ± 0.0008	0.0066 ± 0.0004	0.0045 *
	GSH (μg GSH/g tissue)	453.6 ± 413.2–555.1	287.9 ± 246.4–405.3	0.0175 *
	LOOH (mmol/mg tissue)	11.25 ± 2.918	26.6 ± 1.134	0.0004 *
	Total protein (µg/mL)	10.15 ± 0.3855 (*n* = 6)	8.19 ± 0.4223 (*n* = 6)	0.0064 *

C—control group; 5-FU—treated group; CAT—catalase; SOD—superoxide dismutase; GST—glutathione S-transferase; GSH—reduced glutathione; LOOH—lipid hydroperoxides. Data are expressed as the mean ± standard error (parametric) and median ± lower limit-upper limit (nonparametric), *n* = 7. * *p* < 0.05.

**Table 4:** Markers of inflammation in the kidneys, liver, and lungs of rats treated with 5-FU.

	Analysis	C	5-FU	*p* Value
Liver	MPO (mD.O./mg protein)	0.1415 ± 0.0096	0.1818 ± 0.0139	0.0347 *
	NAG (mD.O./mg protein)	1.651 ± 0.1065	2.093 ± 0.1348	0.0245 *
	NO (μM/µL)	95.92 ± 10.3	199.6 ± 22.44	0.0012 *
	IL-1β (pg/mL)	4686 ± 585.2	6773 ± 568.9	0.0252 *
	IL-6 (pg/mL)	13,126 ± 1599	7902 ± 1311	0.0266 *
	IL-10 (pg/mL)	118.7 ± 44.02–334.2	174.4 ± 97.41–259.4	0.5350
Kidney	MPO (mD.O./mg protein)	0.1437 ± 0.0098	0,1143 ± 0.0054	0.0215 *
	NAG (mD.O./mg protein)	3.53 ± 0.225	2.847 ± 0.1235	0.0208 *
	NO (μM/µL)	101.2 ± 29.16 (*n* = 6)	127.4 ± 42.229 (*n* = 6)	0.6209
	IL-1β (pg/mL)	10,217 ± 863.9	10,511 ± 789.9 (*n* = 6)	0.8088
	IL-6 (pg/mL)	112,944 ± 10,176–408,206	54,769 ± −12,492–27,669	0.5350
	IL-10 (pg/mL)	1795 ± 504	3112 ± 949.6	0.2441
Lung	MPO (mD.O./mg protein)	0.2656 ± 0.0401	0.4043 ± 0.0223	0.0105 *
	NAG (mD.O./mg protein)	2.618 ± 0.3137	4.999 ± 0.605	0.0044 *
	NO (μM/µL)	17.96 ± 3.816–158.1	235.3 ± 209.8–297.2	0.0006 *
	IL-6 (pg/mL)	1682 ± 380.3	834.9 ± 75.82	0.0495 *

C—control group; 5-FU—treated group; MPO—myeloperoxidase; NAG—N-acetyl-glucosaminidase; NO—nitric oxide; IL-1β—interleukin 1β; IL-6—interleukin 6; IL-10—interleukin 10. Data are expressed as the mean ± standard error (parametric) and median ± lower limit-upper limit (nonparametric), *n* = 7. * *p <* 0.05.

## Data Availability

All materials described in this manuscript, including all relevant raw data, are freely available to any researchers who may wish to reuse or reanalyze them. If any conclusions made in the paper depend on a particular dataset then this dataset will be made available to the readers.
